# Epigenetic Editing in Neurological and Neuropsychiatric Disorders: Pioneering Next-Gen Therapeutics for Precision Gene Control

**DOI:** 10.1007/s12035-025-05590-1

**Published:** 2025-12-28

**Authors:** Hany E. Marei

**Affiliations:** Department of Cytology and Histology, Faculty of Veterinary Medicine, Mansoura University, Mansoura, 35116 Egypt

**Keywords:** Epigenetic editing, Neurological disorders, Precision medicine, Gene regulation, Therapeutic strategies, Next-generation therapies

## Abstract

Epigenetic editing has emerged as a promising approach in the treatment of neurological and neuropsychiatric disorders, enabling the precise and enduring modification of genes associated with these conditions. Interventions that focus on chromatin, such as programmable systems like CRISPR/dCas9, zinc-finger proteins, and TALEs linked to epigenetic effector domains, enable the modification of DNA methylation, histone modifications, and noncoding RNA control at specific loci. This work integrates current progress in understanding the epigenetic landscape of neurological neuropsychiatric disorders, highlighting the functions of DNA methylation (de novo vs maintenance, active versus passive demethylation), histone remodeling, and context-dependent gene regulation. We emphasize that the dysregulation of these processes is essential to diseases such as Alzheimer’s disease (AD), Parkinson’s disease (PD), Huntington’s disease (HD), and major psychiatric disorders. Innovative therapeutic approaches, including KRAB- and TET-based repressors, “hit-and-run” epigenome editing, and targeted noncoding RNA regulation, are analyzed alongside translational methodologies that utilize gene therapy vectors, nanoparticle delivery systems, and inducible expression mechanisms. We also examine proof-of-concept studies that demonstrate how to prevent gene expression and alter the transcriptional networks of diseased cells in living organisms. We identify current challenges, including off-target effects, delivery issues, inadequate understanding of long-term stability, and the need for reliable diagnostics, while highlighting the translational promise of combining epigenetic clearance with biogenesis and repair. This review is aimed at providing a comprehensive and critical examination of the molecular principles, therapeutic strategies, and translational obstacles associated with epigenetic editing in neurological and neuropsychiatric disorders, thereby facilitating the development of next-generation precision therapies.

## Introduction

The development, activity, and plasticity of the nervous system depend critically on epigenetic control. Epigenetic mechanisms, including DNA methylation, histone modifications, nucleosome relocation, higher-order chromatin remodeling, non-coding RNAs (ncRNA), and RNA and DNA editing, play a crucial role in controlling gene expression without altering the underlying DNA sequence. Brain differentiation, synaptic plasticity, and the general preservation of brain networks all depend on these mechanisms. Disturbances in these precisely calibrated epigenetic environments might cause abnormal gene expression patterns, aggravating the pathophysiology of several neurological and neuropsychiatric disorders [1]. 

It is now quite evident how crucial epigenetic dysregulation is to neurological illnesses. Altered epigenetic marks have been linked to conditions including Alzheimer’s disease (AD), Parkinson’s disease (PD), and many neurodevelopmental diseases, hence producing faulty gene expression and neuronal activity. For example, abnormal DNA methylation and histone modification patterns in the brains of persons with these disorders have suggested direct links between epigenetic changes and disease pathophysiology. Moreover, since epigenetic pathways are changeable, this generates optimism for new treatment; safety and efficacy remain issues [2].

These revelations have led the discipline of epigenetic editing to become a potential treatment modality. Epigenetic editing involves the targeted modification of epigenetic markers to restore standard gene expression patterns. Scientists can precisely alter aberrant epigenetic modifications by carefully modifying DNA methylation and histone modifications at specific genomic loci using techniques such as CRISPR-based systems. This accuracy enables the rectification of disease-associated epigenetic aberrations, potentially providing therapies for many neurological disorders. Altering epigenetic information can result in the direct and enduring modification of gene expression linked to diseases, providing a novel avenue for fundamental research on the CNS and therapeutic interventions for psychiatric and neurological disorders [3]. Recent comprehensive reviews outline the rapid evolution of epigenome editors and the translational processes required to advance them toward clinical application [4]. In this review, we explore the emerging field of epigenetic editing as a potential treatment for neurological diseases and neuropsychiatric disorders. We will consider the basic modalities of epigenetic regulation in the nervous system, cell biological mechanisms by which pathology-inducing changes can emerge from altered epigenomic states, and cutting-edge approaches for gene expression control that leverage CRISPR-Cas9-based manipulation of the epigenome, histone modification methodologies, and RNA-based methods. The current challenges and constraints of epigenetic editing, novel techniques for modulating the brain, and precision medicine will also be presented in this review. The review is intended to inform researchers, as well as physicians treating patients with neurological diseases and neuropsychiatric disorders, how epigenetic editing may unseat the current paradigm of treatment and advance causal, disease-modifying therapies by systematically summarizing what we already know and recognizing what we do not.

## Molecular Mechanisms of Epigenetic Regulation

Building on the overview of epigenetic relevance in brain function, the following section explores the molecular mechanisms that govern these regulatory processes. In this context, epigenetic modification is the regulation of gene expression that does not involve changes in DNA sequence. These modifications are critical to managing a cell’s identity, development, and interactions with signals from the environment. From a central epigenetic regulation point of view, dynamic DNA methylation and demethylation processes, as well as histone modifications associated with chromatin remodeling, or participation of ncRNAs in gene expression, are mentioned.

### How DNA Methylation and Demethylation Work

DNA methylation is a fundamental epigenetic change; however, its effects on regulation depend significantly on the context. De novo DNA methyltransferases (DNMTs) such as DNMT3A and DNMT3B are responsible for establishing methylation by adding new methylation marks during development or in response to environmental stimuli. DNMT1, on the other hand, is the primary maintenance methyltransferase, accurately copying existing methylation patterns during DNA replication [5, 6]. This distinction is vital, as the disruption of de novo activity can modify cellular identity, while abnormalities in maintenance methylation undermine epigenetic memory, both of which are relevant to neurological disorders.

The ten-eleven translocation (TET) mediated pathways are especially prevalent in the brain, where 5-hydroxymethylcytosine accumulates and influences activity-dependent transcription. However, within gene bodies or specific enhancer regions, methylation may be associated with active transcription, suggesting a more complex role than previously acknowledged [7, 8]. This complexity is especially relevant in neurodevelopment and disease, where aberrant methylation may either suppress protective genes or incorrectly activate deleterious pathways. Understanding these tasks is crucial for developing accurate epigenetic editing strategies to restore healthy methylation patterns. Passive or active mechanisms can lead to loss of DNA methylation. Passive demethylation occurs indirectly during DNA replication due to insufficient maintenance of methylation. Active methylation involves oxidation of 5-methylcytosine by TET proteins and base excision repair.

Within mostly CpG dinucleotides, DNA methylation adds a methyl group to the 5-carbon of cytosine residues. The dynamic interaction between methylation and demethylation underlies processes such as embryonic development, X-chromosome inactivation, and genomic imprinting [9]. On the other hand, histone deacetylases (HDACs) remove these acetyl groups, leading to transcriptional suppression. The specific amino acid residue changed, and the number of methyl groups added determines whether histone methylation activates or represses transcription. Chromatin remodeling complexes, which are part of the Switch/Sucrose Non-Fermentable (SWI/SNF) family, use the hydrolysis of adenosine triphosphate (ATP) to rearrange nucleosomes. This changes how easily DNA can be accessed by transcriptional machinery. Histone modifications and chromatin remodeling ensure precise regulation of gene expression patterns essential for cellular function and identity [10]. 

There are many epigenetic processes that make ncRNAs essential for controlling gene expression. lncRNAs influence gene expression, histone modifications, and DNA methylation by attracting chromatin-modulating complexes to specific genomic loci. HOX transcript antisense RNA (HOTAIR), a long non-coding RNA (lncRNA), interacts with polycomb repressive complex 2 (PRC2) to suppress the transcription of particular genes. They usually attach to complementary sequences in mRNAs, miRNAs, and small ncRNAs, and they either break down or slow down translation. Along with post-transcriptional control, miRNAs also target DNMTs and demethylases, which control DNA methylation and gene expression at the epigenetic level. Multilevel gene expression control makes ncRNAs more complicated [11].

**Fig. 1:**
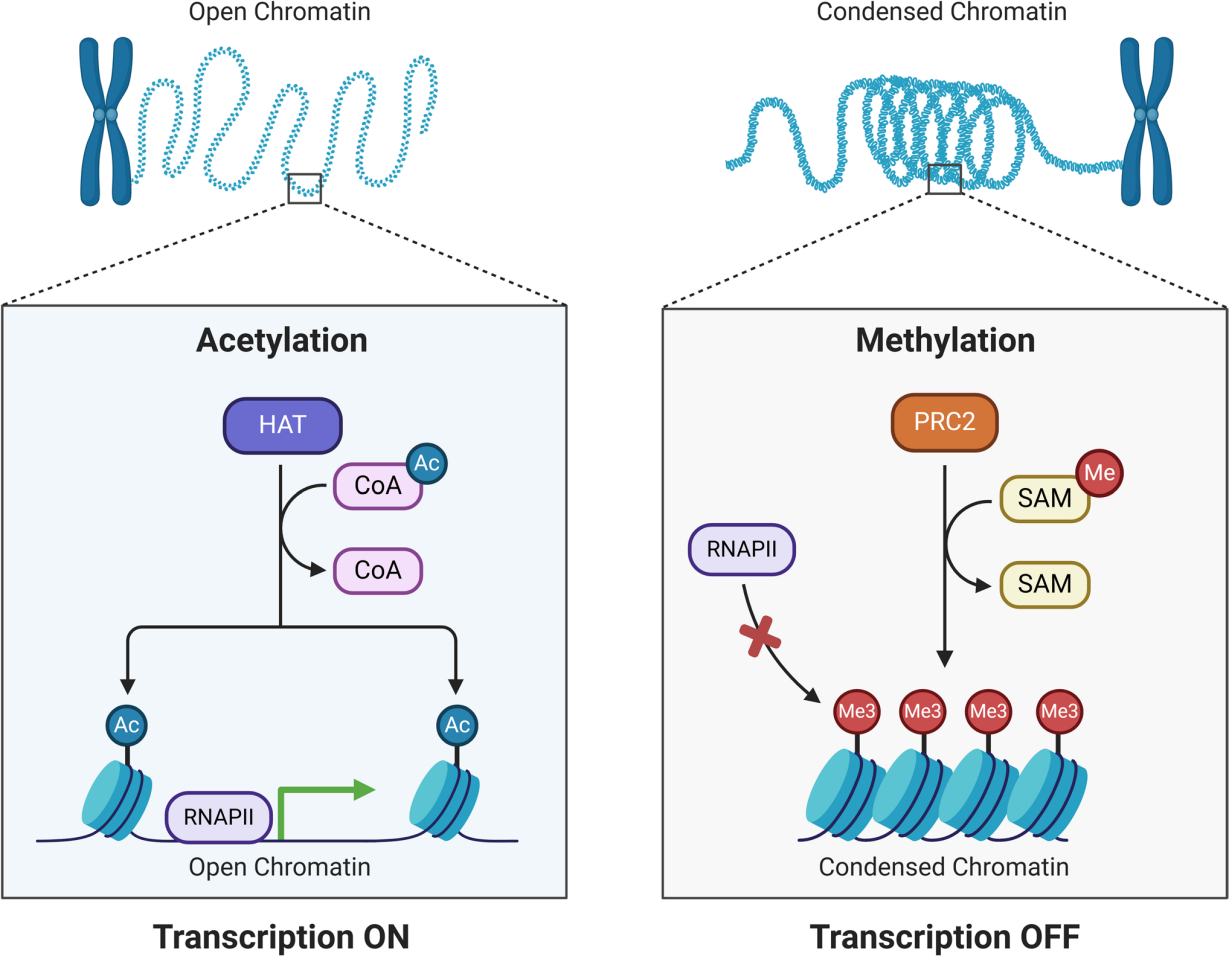
Epigenetics and gene expression. This diagram shows how epigenetic changes relate to the control of gene expression. Shown as genetic material, the DNA strand has coding and non-coding sections. Regulation of gene expression depends critically on epigenetic changes, including DNA methylation and histone modifications (like acetylation and methylation). Usually, by inhibiting transcription factor binding, DNA methylation silences genes; histone changes either relax or compact chromatin structure, affecting gene accessibility. Relaxed chromatin binds transcription factors and RNA polymerase, facilitating mRNA synthesis, whereas compacted chromatin results in epigenetic marks that block transcription factors, suppressing gene expression. Stress or food are environmental elements that can cause epigenetic changes affecting gene expression, so establishing a dynamic feedback loop whereby gene expression can influence epigenetic modifications underlines the reversible and dynamic character of this control [10]

Identical biochemical markers exhibit distinct transcriptional effects contingent upon locus and cellular state, necessitating systematic, locus-specific validation in therapeutic development [12]. The epigenetic changes, such as DNA methylation and histone modifications, regulate gene expression by altering the chromatin structure and the availability of transcription factors. DNA methylation typically silences gene expression, but histone modifications can either initiate or inhibit transcription. Whether chromatin relaxes or compacts affects gene expression (Fig. 1).

## Epigenetic Editing Tools: A Technological Overview

**Fig. 2:**
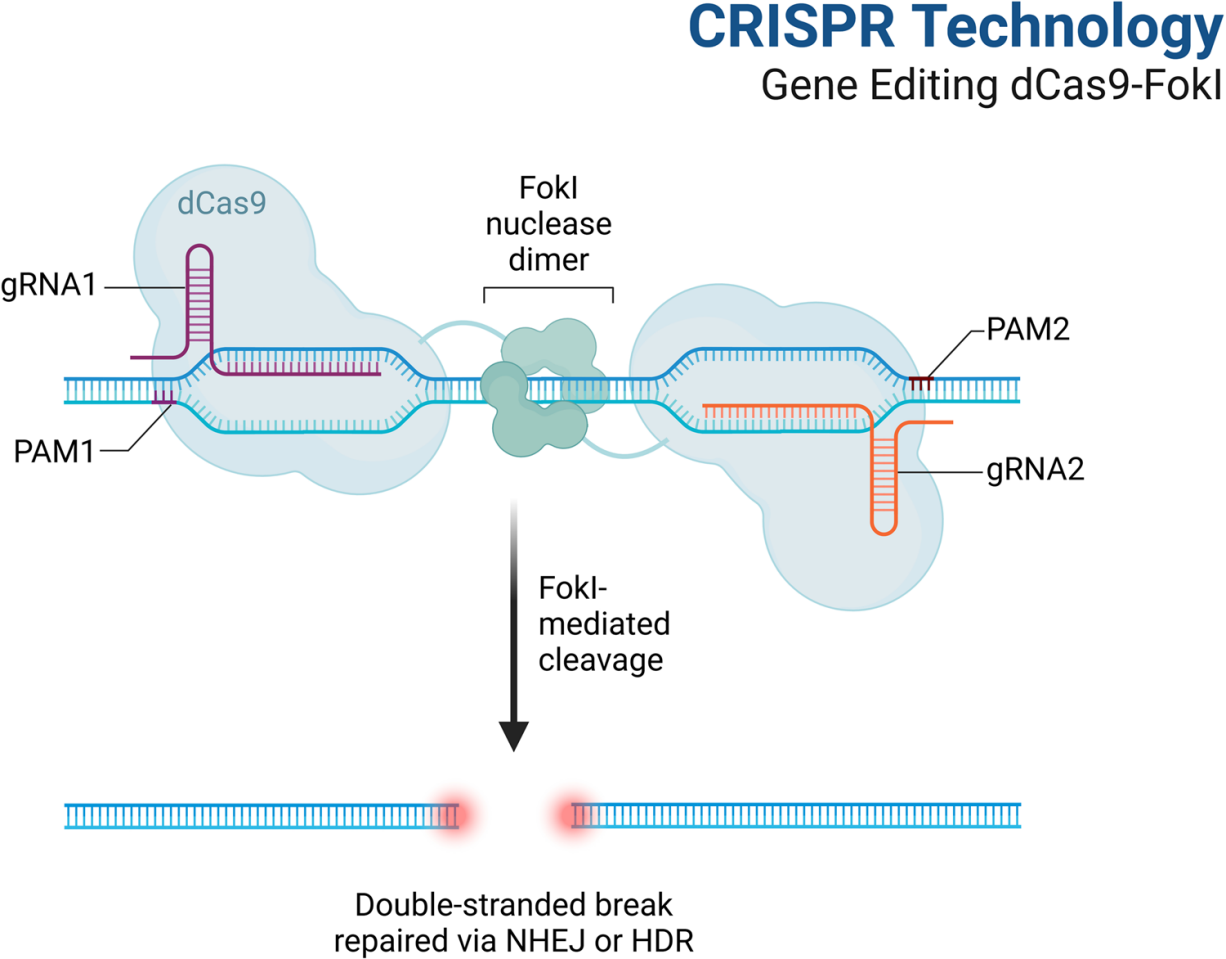
**CRISPR **technology: gene editing dCas9-FokI. This image displays the CRISPR-based gene editing system utilizing dCas9-FokI. The Cas9 protein has been changed into dCas9, which stands for “dead Cas9.” This version of the protein lacks the nuclease activity required to cut DNA. Instead, it is linked to the FokI nuclease, an enzyme that only functions when it pairs with two specific guide RNA (gRNA) molecules. The dCas9-FokI fusion protein cuts the genome at the location specified by the gRNA. When the dCas9-FokI fusion protein is lined up correctly with two target sites next to each other, the FokI nucleases only break one strand of DNA. Depending on the type of repair template or proteins used, this targeted DNA cleavage can be utilized for various purposes, such as gene knockout, chromatin modifications, or targeted gene repair. This method allows for precise gene editing, enabling changes to the genome without the issues associated with other CRISPR-Cas9 systems [13]

Having established the foundational mechanisms of epigenetic regulation, we now turn to the technologies developed to manipulate these processes precisely. This technology review will discuss the fundamentals of epigenetic editing, key editing platforms, and their clinical and research applications. Unlike CRISPR-Cas9, which represents the traditional gene editing technology aimed at inducing permanent alterations to the DNA sequence (Fig. 2), epigenetic editing enables precise control of gene expression. It is therefore expected to provide an effective tool for treating a wide array of complex diseases, including neurological and neuropsychiatric illnesses.

### CRISPR-Based Epigenetic Editors

Epigenetic editing based on CRISPR has changed the way genes are controlled by allowing precise changes to the epigenome without changing the DNA sequence itself. This method uses a catalytically inactive Cas9 protein (dCas9) and different epigenetic modifiers to modify gene expression very precisely by adding specific epigenetic marks [7, 14].

The dCas9-TET fusion protein directs the TET enzyme to specific genomic locations, enabling the oxidation and subsequent elimination of methyl groups, thereby reactivating repressed genes [15]. Targeted TET-based demethylation techniques (dCas9–TET1CD) can reactivate methylation-silenced loci; however, studies highlight locus variability and the potential for collateral demethylation, necessitating allele- or locus-specific confirmation [16].

Alternatively, dCas9 and DNMTs like DNMT3A or DNMT3B can concentrate on DNA methylation [17]. CRISPR-based systems can also change histone proteins, which are proteins that control how genes are expressed and how chromatin is structured [18], and neurological disorders [19]. Notably, the programmable dCas9–p300 acetyltransferase provided the initial compelling evidence that locus-targeted histone acetylation (H3K27ac) is sufficient to activate endogenous enhancers and promoters, thereby establishing a functional framework for transcriptional reactivation in neuronal systems [20]. 

CRISPR-based epigenetic editors can study complicated networks of gene regulation and make treatments that work. Scientists can separate the effects of gene expression from changes in epigenetics by precisely adding or removing epigenetic marks. These devices also facilitate epigenome-targeted therapy by rectifying aberrant epigenetic states associated with specific diseases. Initial CRISPR-mediated epigenetic editing trials for sickle cell anemia [21] and beta-thalassemia [22] demonstrate encouraging results that may be extrapolated for broader therapeutic applications. CRISPR-based epigenetic editors, such as dCas9-TET for DNA demethylation, DNMT3A/B for targeted DNA methylation, and p300 and LSD1 for histone modifications, enable precise control of the epigenome. These technologies hold promise for therapeutic applications that correct epigenetic aberrations in disease states, significantly expanding our understanding of gene control.

In CRISPR-Cas-mediated gene regulation, the Krüppel-associated box (KRAB) domain is a potent and common transcriptional repressor. When KRAB is linked to catalytically inactive Cas9 (dCas9), it recruits the co-repressor KAP1 (TRIM28), which subsequently recruits heterochromatin effectors, such as HDACs and H3K9 methyltransferases. This process stops gene expression, even from enhancers that are several kilobases away, and makes heterochromatin form in some places [23–25]. KRAB-dCas9 is a part of CRISPRi systems that work well and are used a lot to turn off genes. It is commonly utilized in functional genomics and screening, and when influenced by doxycycline, it offers controllable functionality that can be activated or deactivated [26, 27]. Systematic investigations have demonstrated that silence following KRAB-dCas9 recruitment is significantly influenced by locus characteristics and associated effectors, with combinations such as KRAB in conjunction with DNMT3A/3L or MeCP2 creating conditions conducive to enduring repression [28–30].

The combination of KRAB and TET domains is not commonly documented; however, specific sophisticated CRISPR epigenome-editing systems incorporate both repressive and demethylating components, either in succession or via modular recruitment, to dynamically modulate gene expression—utilizing KRAB for intense repression and TET for accurate reactivation. For example, multi-domain techniques that utilize KRAB-MeCP2 fusions have demonstrated long-lasting silencing that is not affected by methylation, and TET fusions function as additional epigenetic activators [31, 32].

### TALEN and ZFN in Epigenetic Modulation

Two creative technologies that have broadened the toolkit for focused genome editing are TALEN (transcription activator-like effector nucleases) and ZFN (zinc finger nucleases). TALENs comprise a DNA-binding domain derived from transcription activator-like effectors (TALEs) and a nuclease domain derived from the FokI restriction enzyme [33].

ZFNs are artificial proteins that have FokI nuclease domains and zinc finger motifs that can bind to DNA sequences. ZFNs, like TALENs, can be changed by epigenetic enzymes and cause double-stranded DNA breaks. Targeted DNA methylation is a promising use for ZFNs. You can target genomic DNA methylation by fusing ZFNs with DNMT3A or DNMT3B. Controlling gene expression has allowed this method to silence genes of interest in model systems accurately [34]. ZFNs can also be linked to enzymes that change histones, like p300, to turn on gene expression at specific locations by selectively acetylating histones. ZFNs modify histone tails, altering chromatin structure and accessibility, which in turn impact gene expression [35].

TALENs and ZFNs are better than RNA interference (RNAi) and small-molecule inhibitors at changing gene expression because they are precise and have long-lasting effects. TALENs and ZFNs can alter the epigenome with great precision because they can target specific chromatin locations with high specificity. This is important for understanding how genes are controlled in many diseases. For instance, a hallmark of cancer is the abnormal methylation of oncogenes or tumor suppressor genes. A possible treatment strategy to alter gene expression and halt disease progression could involve directly modifying DNA methylation at these loci using TALENs and ZFNs [36]. Moreover, epigenetic regulation by TALENs and ZFNs has shown promise in neurological diseases, where alterations in DNA methylation and histone modifications are associated with the onset and progression of disorders such as AD and PD [30], [37].

### RNA-Based Epigenetic Modulators

**Fig. 3:**
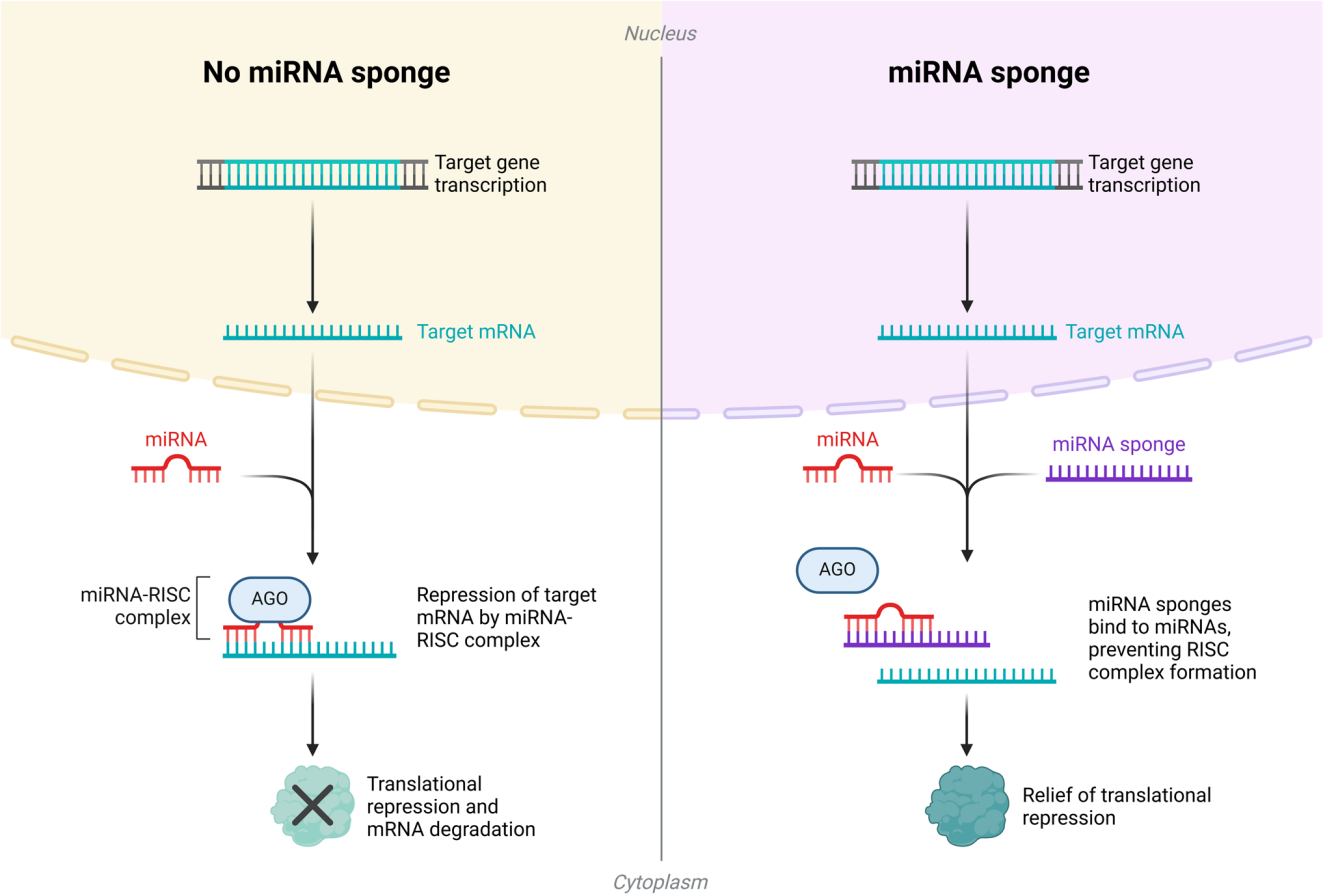
miRNA activity suppression with miRNA sponges. This diagram shows how miRNA activity can be decreased by using miRNA shields. miRNA sponges are artificial RNA molecules that are made to have many binding sites that are the same as those on specific miRNAs. When this miRNA sponge is introduced into cells, it binds to the target miRNA(s) and prevents them from interacting with their standard mRNA targets. By binding to free miRNAs, the sponge lowers their number. This prevents the RNA-induced silencing complex (RISC) from silencing genes. This method can be used to learn more about how specific miRNAs work, to restore the production of genes that miRNAs target, or to change the way genes are expressed. As a result, genes that would usually be silenced by the miRNA become more active. This makes it possible to study how miRNAs control different biological processes [40]

RNA-based epigenetic modulators are a new way to change the epigenetic landscape directly or indirectly, which gives them control over gene expression. This part explains how RNA-based epigenetic modulators like lncRNAs, Small Interfering RNAs (siRNAs), microRNAs (miRNAs), and RNA-guided CRISPR systems work, how they can be used, and how they might be used in therapy (Fig. 3). lncRNAs, which control gene expression and chromatin states, have been the focus of recent research [38]. Other lncRNAs, such as HOTAIR, have been shown to interact with chromatin-modifying complexes and facilitate the spread of repressive chromatin marks, thereby silencing target genes [39]. Because they can be developed to primarily target and control gene expression by altering the epigenome, lncRNAs represent a promising class of RNA-based modulators with therapeutic potential.

siRNAs and miRNAs are another type of RNA-based epigenetic modulator that play a crucial role in gene regulation. They affect both transcription and post-transcriptional events. siRNAs are double-stranded RNA molecules that start the RNAi pathway. This pathway stops target mRNA molecules from being turned into proteins, which breaks them down. Recent studies indicate that siRNAs, in addition to their established role in post-transcriptional gene silencing, may also affect the epigenetic state of the genome by facilitating the formation of repressive chromatin marks [41]. Chromatin remodeling, histone modification, and DNA methylation are among the several epigenetic processes whose control has been linked to miRNAs. For instance, miR-29 family members have been demonstrated to target DNMTs [42]. Hence, miRNAs control DNA methylation. By targeting histone-modifying enzymes, such as HDACs, miRNAs can thus control histone modifications, influencing the chromatin state and gene expression [43]. 

**Fig. 4:**
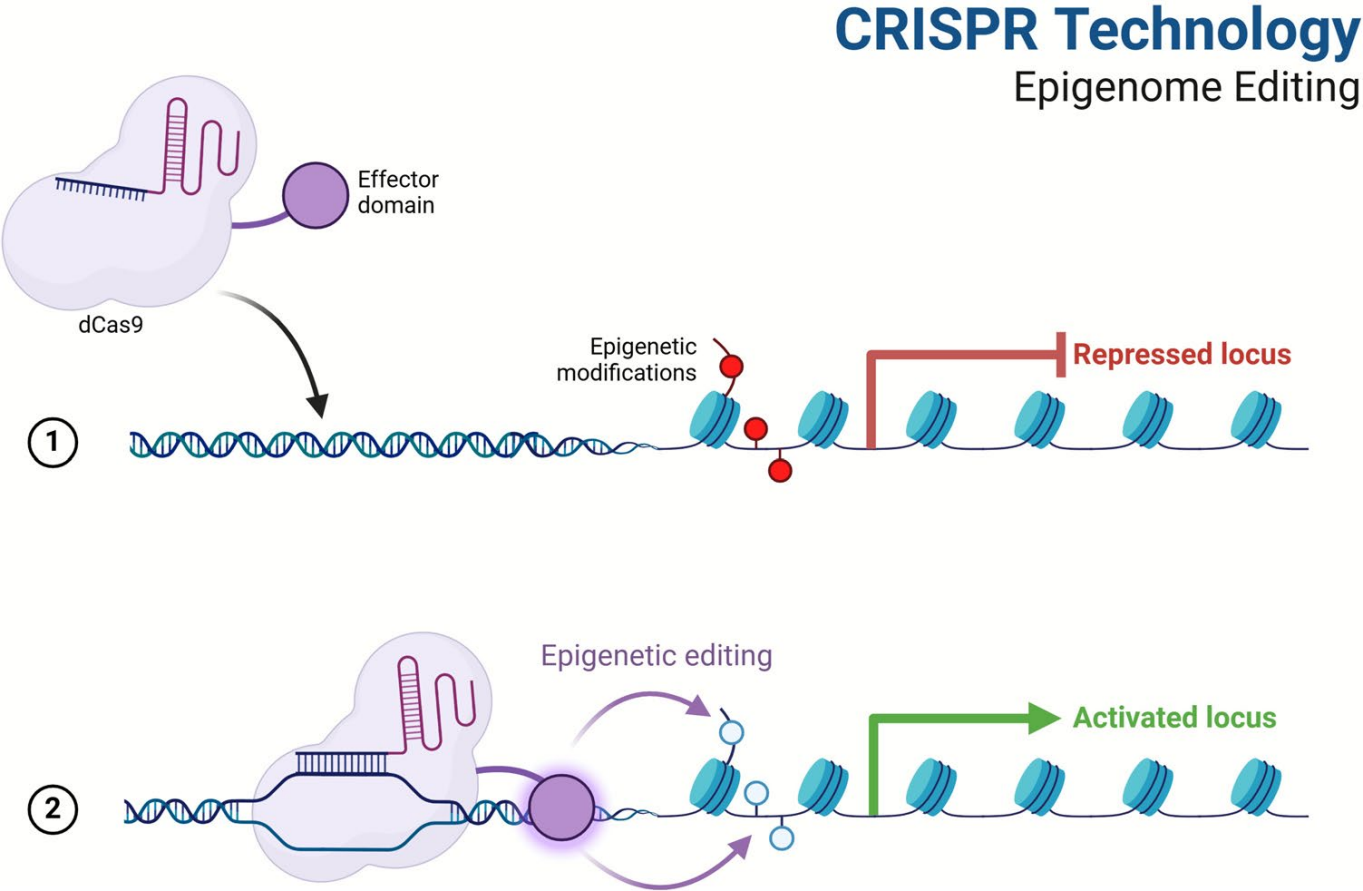
CRISPR technology: epigenome editing. This image illustrates how CRISPR-based technology for editing the epigenome functions. It makes minimal changes to epigenetic marks to control gene expression without altering the underlying DNA code. The system utilizes a Cas9 protein known as dCas9. It is combined with enzymes that change the way genes are expressed, such as DNMTs or histone-modifying enzymes. The gRNA instructs the dCas9 fusion protein on where to direct its action in the genome. There, the epigenetic enzyme attaches and chemically modifies the DNA or histones by adding or removing methylation or acetylation. These changes alter the shape of chromatin, which can influence whether genes are turned on or off. This method lets specific changes be made to gene function, which makes it easier to study gene regulation, disease processes, and possible therapeutic interventions. The original genomic sequence is also kept intact

RNA-guided CRISPR systems have changed the field of gene editing and opened up new ways to change genes in a specific way through epigenetic control (Fig. 4). For example, it has been shown that combining dCas9 with the TET1 DNA demethylase domain causes DNA demethylation at certain places, which turns on genes that were turned off before [44]. In the same way, dCas9 can be linked to DNMT3A or DNMT3B to speed up DNA methylation at certain places in the genome, which can turn off genes [45].

RNA-based epigenetic modulators have tremendous therapeutic potential, particularly in disorders where epigenetic dysregulation is a contributing cause. In cancer, for example, the dysregulation of DNA methylation and histone modifications is essential for tumorigenesis—that is, for silencing tumor suppressor genes or activating oncogenes. Designed lncRNAs or siRNAs targeting DNMTs or other RNA-based epigenetic modulators provide the means of reprogramming the epigenome to restore standard gene expression patterns. Similarly, in neurological and neuropsychiatric disorders where epigenetic modifications are associated with the initiation and advancement of diseases like AD and PD, RNA-based epigenetic regulation may present innovative therapeutic strategies by counteracting detrimental epigenetic alterations. Targeting specific epigenetic pathways, recent investigations have demonstrated that RNA-based treatments can restore the expression of genes associated with synaptic plasticity and neuronal survival [46]. Their targeted and regulated delivery through nanoparticles or viral vectors augments the therapeutic potential of RNA-based modulators for clinical applications. Finally, RNA-based epigenetic modulators—such as lncRNAs, siRNAs, miRNAs, and RNA-guided CRISPR systems—represent a new frontier in the control of gene expression through epigenetic means. These RNA molecules provide highly targeted and dynamic approaches for modifying the epigenome, which could have significant effects on how diseases are treated. Where epigenetic changes contribute to disease pathogenesis, RNA-based epigenetic modulation technologies have enormous potential for treating various diseases, including cancer, neurological disorders, and neuropsychiatric diseases. The translation of RNA-based epigenetic modulators into clinical applications will rely on forthcoming studies to improve their delivery, specificity, and stability [47]. 

## Applications of Epigenetic Editing in Neurological Disorders

### Neurodevelopmental Disorders

With the core tools outlined, it is essential to examine how these editing systems have been applied to model and potentially treat neurological and neuropsychiatric disorders. Targeting the epigenome to precisely and reversibly control gene expression has transformed epigenetic editing for treating neurological diseases [48], [49]. Rett syndrome is a neurodevelopmental disorder (X-linked dominant) caused mainly by mutations in methyl-CpG-binding protein 2 (MECP2). Most clinical trials for Rett syndrome are still in their early stages of development. TSHA-102, an AAV serotype 9-based vector that delivers functional MECP2, is being tested in a gene therapy investigation (NCT04181723) as the first human application to restore MECP2 function [50]. Another trial (NCT05060123) is looking into AAV-based therapy NGN-401 with a MECP2 transgene for dose management [51]. These studies mainly focus on gene replacement rather than direct modification of epigenetics; yet, they demonstrate the feasibility of altering MECP2 expression in a therapeutic context. Future therapeutic applications of CRISPR/dCas9-TET or KRAB-based epigenetic editing techniques may leverage the safety and delivery insights gained from these ongoing studies.

FXS is another neurodevelopmental disorder that makes it hard for people to think, act, and get along with others [30]. Targeting the epigenetic regulation of Fragile X Messenger Ribonucleoprotein 1 (FMR1) has enabled the gene to be reactivated, hence restoring FMRP expression in neurons [31]. In models of FXS, DNA demethylation of the FMR1 region has been shown to offer a potential therapeutic approach for patients [32]. This will help restart transcription. In addition to CRISPR-based systems, other epigenetic editing technologies, including histone modifiers and DNMTs, have been utilized to modify the epigenome in neurodevelopmental disorders. For instance, paired with DNMT enzymes, CRISPR/Cas9 systems have been found to add DNA methylation marks at specific genomic loci, therefore providing a tool to repress or silence genes active in neurodevelopmental processes [34]. Conversely, demethylases such as TET1 can be employed to remove DNA methylation and re-activate quiet genes, including MECP2 in Rett syndrome and FMR1 in Fragile X Syndrome (FXS) [37]. Targeting histone modification enzymes—including histone acetyltransferases (HATs) and HDACs—helps to alter chromatin structure and gene expression in these disorders. It offers hope for future therapeutic strategies, as it allows one to either minimize or reverse the effects of neurodevelopmental disorders.

FXS is a neurodevelopmental disorder marked by cognitive deficits, behavioral irregularities, and social challenges. An elevated CGG repeat in the 5' untranslated region of the FMR1 gene leads to promoter hypermethylation, transcriptional silencing, and the lack of FMRP. FMRP is a protein that binds to RNA and is essential for synaptic plasticity and neuronal transmission. The reactivation of FMR1 in patient cells restores FXM and maintains essential cellular characteristics, indicating that epigenetic silencing may serve as a potential therapeutic target [52–54]. Initial proof-of-concept demonstrated that global DNA demethylation using 5-aza-2′-deoxycytidine can reactivate FMR1 in FXS lymphoblastoid cells; however, this approach faces significant translational challenges due to its lack of specificity for particular loci [52]. Recently, targeted epigenetic editing utilizing CRISPR/dCas9 and the TET1 demethylase has effectively demethylated the FMR1 promoter/CGG region and reactivated endogenous FMR1 in neurons derived from human FXS-induced pluripotent stem cells (iPSCs), simultaneously restoring aberrant electrophysiological characteristics. Additional research indicates that the combination of initial demethylation with small molecules that modify chromatin may extend the duration of FMR1 expression in neurons, mitigating concerns regarding stability following the removal of the editing stimulus [54]. Researchers are investigating RNA-guided techniques that collaboratively activate chromatin at the FMR1 gene to reinitiate transcription. These strategies integrate CRISPRa fusions with HATs or transcriptional activators, combined with ASO/siRNA techniques that target specific transcripts or chromatin structures [53–55]. 

In addition to CRISPR-TET–mediated demethylation, a broader array of epigenetic editors has been developed and employed in models of neurodevelopmental disorders. Catalytically inactive Cas9 can be utilized in conjunction with epigenetic effectors, such as DNMTs (for targeted repression) or demethylases (for targeted activation), as well as histone modifiers (including acetyltransferases or deacetylases), to precisely introduce or eliminate modifications at specific loci. In neurological research, similar approaches have been employed to either suppress the activity of disease-associated genes by introducing methyl groups or to demethylate nonfunctional genes, specifically MECP2 and FMR1, in cellular models of Rett syndrome and FXS, respectively [53]. Precision epigenome editing and X-chromosome reactivation (XCR) approaches aim to enhance the functionality of the intact MECP2 allele on the inactive X chromosome in females who are heterozygous for the gene. A recent translational study from mouse to human demonstrated that multiplex epigenome editing can alter chromatin at X-linked targets and partially enhance disease-relevant phenotypes, underscoring both the potential and the safety concerns (e.g., risks of MECP2 over-expression toxicity and mosaic XCR) that must be addressed before clinical application [56]. 

The clinical application of genuine epigenetic editing (i.e., programmable and locus-specific writers/erasers) in FXS or Rett syndrome has not commenced; however, similar gene-regulatory methodologies have advanced to human trials, demonstrating the feasibility of targeting epigenetic states in neurodevelopmental disorders. Angelman syndrome (AS) results from the epigenetic silencing of the paternal ubiquitin protein ligase E3A (UBE3A) allele in neurons. Rugonersen/RO7248824 is an antisense oligonucleotide (ASO) that selectively targets the UBE3A-ATS transcript. Initial studies have shown that it is both practical and safe for children [57, 58]. The gene-regulatory treatment TSHA-102, based on AAV9, is currently in Phase 1/2 clinical trials for the treatment of Rett syndrome. A regulated expression cassette reduces MECP2 overexpression. Teens and adults (REVEAL Adult; NCT05606614) and children (REVEAL Pediatric; NCT06152237) are studied. These are not true epigenetic editors, but they provide methods for therapeutically administering CNS drugs, analyzing their effects, and establishing protocols for future FXS and Rett Syndrome epigenetic editing therapies. Still, CNS epigenetic editing concerns need to be resolved: (i) administering it extensively to the human brain and spinal cord (most preclinical studies use viral vectors for ex vivo or localized in vivo delivery); (ii) ensuring it only targets the right chromatin (reducing off-target chromatin remodeling); (iii) ensuring it lasts (sustaining corrected epigenetic states through cellular divisions and neuronal activity); and (iv) ensuring it is safe, including immunogenicity, precise dosage regulation.

Preclinical investigations and initial clinical experiences with CNS-targeted ASOs and AAV-mediated gene regulation establish a basis for tackling these challenges through comprehensive pharmacological assessment, biodistribution analysis, and extended surveillance [53, 56, 59]. Epigenetic alteration remains a feasible approach for the application of precision medicine in the treatment of neurodevelopmental disorders. It specifically targets the detrimental epigenetic state, rather than the DNA sequence, and exhibits locus specificity not present in global epigenetic therapies. It can also change the course of a disease by either reactivating it or suppressing it in vivo, depending on how well and for how long it is given.

### Neurodegenerative Diseases

Epigenome editing is a novel and powerful approach to treating neurodegenerative diseases (NDDs). Epigenetic modifications influence cellular degeneration and gene expression, playing a role in neurodegenerative disorders such as AD and PD.

Targeting epigenetic marks at genomic loci modulates genes associated with neuroprotection, neuronal survival, and disease progression, elucidating the concept of epigenome editing in NDDs. Epigenome editing precisely and reversibly modulates gene activity without altering the DNA sequence. Epigenome editing typically utilizes catalytically inactive Cas9 (dCas9) in conjunction with epigenetic modifiers, including DNMTs, TET enzymes, HATs, or HDACs. These methods regulate gene expression by modifying epigenetic markers, such as DNA methylation and histone modifications.

The APOE gene is the primary focus of research for epigenome editing in AD [36]. Epigenetic modifications may elevate BDNF expression in AD, mitigating neuronal damage and enhancing cognitive function. dCas9-p300, a histone acetylation fusion protein, has been shown to raise BDNF levels and enhance synaptic activity in models of AD [37]. PD is characterized by the aggregation of α-synuclein; consequently, modulation of its expression has been recognized as a potential therapeutic approach. In cellular models of PD, targeted use of dCas9-DNMT3A (a DNA methyltransferase) has been shown to lower α-synuclein expression by raising DNA methylation at its promoter. This lowers α-synuclein aggregation and toxicity [38]. On the other hand, epigenetic activation by CRISPR/dCas9-p300 may turn on helpful genes that lower α-synuclein toxicity, like the DJ-1 gene, which would make PD models more neuroprotective—promising preclinical evidence on NDD epigenetic editing. Animal models of AD have shown improved cognitive function and reduced amyloid plaque formation through CRISPR-based epigenome editing, targeting key genes involved in neuroprotection and synaptic plasticity [39]. Epigenetic editing has also shown a reduction in α-synuclein aggregation and motor impairment in PD models, suggesting the potential of this approach to slow down the course of disease. Translating these preclinical successes into practical applications remains a challenge for us, though. Particularly for NDDs, the distribution of epigenetic editing tools to the brain must overcome significant challenges related to blood–brain barrier (BBB) penetration, off-target effects, and long-term safety [41]. Several preclinical studies and ongoing clinical trials are aimed at demonstrating the feasibility of epigenome editing for the treatment of NDDs. Although most of this research is still in its early stages, it indicates that epigenetic ideas are making significant progress toward being applied in medicine. Clinical research utilizing CRISPR-Cas9-based gene editing has demonstrated considerable promise for genetic disorders, such as sickle cell anemia; analogous investigations for AD and PD may ensue in the forthcoming years. To make epigenome editing tools usable in clinical settings for neurological diseases, it is crucial to enhance delivery methods, such as AAV vectors or nanoparticles. Epigenome editing holds considerable promise as a treatment for NDDs, as it can target key genes linked to disease causation and protect neurons. Although the field is still young, advances in epigenetic modifiers and CRISPR/Cas9 technologies offer promising opportunities to treat challenging diseases, such as AD and PD. Together with preclinical and clinical studies, continuous research of the molecular mechanisms of epigenome editing will help to ascertain the effectiveness and safety of these approaches for human patients.

Epigenome editing has not yet advanced to clinical trials for neurodevelopmental or NDDs; nevertheless, recent clinical experiences with in vivo and ex vivo CRISPR–Cas9 genome editing provide substantial translational precedents. For example, in vivo editing of transthyretin amyloidosis (NTLA-2001) demonstrated good systemic transport and initial safety/efficacy [60]. Conversely, ex vivo hematopoietic stem cell editing demonstrated lasting advantages in sickle cell disease and β-thalassemia [54]. These tests set the rules for how epigenome editors must work and what they can and cannot do. The development of fundamental tools includes dCas9-p300 for regulated histone acetylation [33], dCas9-DNMT3A for customizable DNA methylation [16], and dCas9-TET1 for locus-specific demethylation [52]. These platforms have enabled proof-of-concept disease modification in neuronal systems, encompassing the reactivation of FMR1 in neurons derived from patients with FXS [54], the enhancement of neuroprotective BDNF signaling [61], and the suppression of pathogenic α-synuclein expression in PD models [59, 62]. Progress in translation will depend on safe and efficient delivery to the CNS using stereotactic, intrathecal, or modified AAV/nanoparticle techniques, as well as comprehensive assessment of off-target effects, duration, and immunogenicity. These findings indicate that, even in the absence of initiated NDDs’ research, the clinical frameworks and regulatory precedents established by genome editing trials are facilitating the impending application of targeted epigenome editing within the CNS [63]. A recent in vivo study notably demonstrated durable, hit-and-run epigenome editing in mice, effectively silencing proprotein convertase subtilisin/kexin type 9 (Pcsk9) and reducing circulating cholesterol through transient epigenetic editors [64].

### Neuropsychiatric Disorders

Epigenetic editing has the potential to address neuropsychiatric disorders such as schizophrenia and major depressive disorder. Environmental and genetic factors that affect the epigenome can contribute to various diseases that exacerbate synaptic plasticity. Recent studies have demonstrated that ncRNA regulation, histone modifications, and epigenetic changes, including DNA methylation, may contribute to the etiology of various diseases. Therapeutic research on epigenetic mechanisms influencing synaptic plasticity-related genes such as BDNF appears promising. BDNF is essential for synaptic remodeling, which affects memory, learning, brain function, and other cognitive processes.

The study concentrates on the epigenetic modification of BDNF and glutamatergic transmission genes. Studies on DNA methylation and histone modification of the BDNF gene indicate that individuals with schizophrenia may synthesize reduced levels of BDNF [37]. Recent advancements in CRISPR/Cas9-mediated epigenetic editing enable the precise activation or repression of genes, indicating potential for therapeutic interventions [42]. Correcting epigenetic markers at specific locations could help restore suitable synaptic plasticity and alleviate symptoms of schizophrenia.

Major depression, another prevalent neuropsychiatric disorder, has been associated with reduced synaptic plasticity gene expression. The use of patient-derived iPSCs has enhanced the study of neuropsychiatric disorders within the framework of epigenetic regulation [45]. This methodology facilitates the examination of disease-relevant cell types, including dopaminergic or glutamatergic neurons, where epigenetic modifications can be manipulated and analyzed. These models also facilitate the identification of new therapeutic targets for epigenetic treatments and aid in the development of more personalized therapy strategies.

Although high-precision contemporary technologies, such as CRISPR-based epigenetic editing, still face challenges in delivering efficacy to specific brain locations and potential off-target consequences [46], we also need more research on how these kinds of treatments affect behavior and brain function over time [42].

Epigenetic editing is a new way to treat neuropsychiatric disorders like schizophrenia and severe depression. Recent translational studies have highlighted the therapeutic potential of epigenetic editing in neuropsychiatric disorders, demonstrating the application of contemporary clinical methodologies. Clinical trials are evaluating HDAC inhibitors, including vorinostat (NCT03018246) and valproic acid (NCT00282436), as adjunctive therapies for schizophrenia and depression, owing to their capacity to modulate gene expression associated with brain plasticity (NCT03018246 and NCT00282436). Simultaneously, preclinical studies utilizing CRISPR/dCas9-p300 epigenome editing have shown the activation of BDNF and glutamatergic signaling genes, effectively alleviating synaptic impairments in animal models of severe depressive disorder [65]. iPSCs-derived neuronal models from individuals with schizophrenia and depression have confirmed abnormal DNA methylation patterns at synaptic plasticity loci, establishing disease-relevant platforms for customized treatment evaluation. Preliminary epigenetic therapies are investigating the targeting of ncRNAs, such as miR-132 and miR-134, which modulate dendritic spine density and synaptic remodeling, and are associated with the pathogenesis of both depression and schizophrenia [66]. The growing body of preclinical and clinical evidence suggests that epigenome editing, in conjunction with traditional pharmaceutical epigenetic modulators, may revolutionize therapeutic approaches for neuropsychiatric disorders.

### Spinal Cord Injury and Regeneration

Spinal cord injury (SCI) is a terrible condition that can cause permanent neurological damage and impairment, which makes life less enjoyable. Because the CNS does not heal very well, SCI usually causes loss of motor, sensory, and autonomic function below the level of the injury. The central nervous system (CNS) of mammals can heal itself, but glial scars and myelin waste make this process harder. SCI makes it more difficult to access medical help. In SCI models utilizing iPSC-derived NS/PCs, axonal regeneration, reduced tissue damage, and enhanced functional recovery have been observed. The efficacy of this procedure depends on the origin and quality of the transplanted cells, as well as the ability to regulate their environment and remove inhibitory factors at the injury site.

Epigenetic regulation is crucial for development, differentiation, repair of damage, gene expression, and cellular function. In SCI, epigenetic mechanisms, including DNA methylation, histone modifications, and ncRNAs, govern both native and transplanted neural stem/progenitor cells (NS/PCs). Recent studies have shown that epigenetic factors affect the efficacy of iPSC-derived NS/PCs in treating spinal cord injury (SCI). Yang et al. (2024) examined the role of epigenetic regulation in enhancing cellular regeneration [48].

**Fig. 5:**
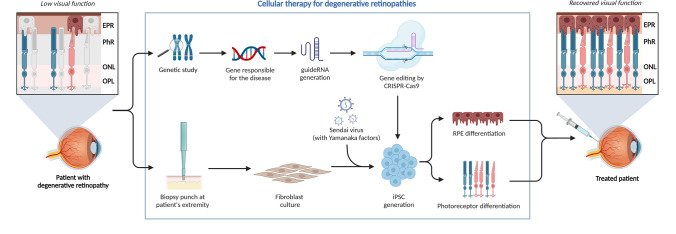
Cellular therapy for degenerative retinopathies. This diagram illustrates the mechanism of cellular treatment for degenerative retinopathies. The goal is to restore vision by transplanting or stimulating cells that can repair damaged retinal tissue. This method usually uses stem cells, such as iPSCs or retinal progenitor cells. You can turn these cells into different kinds of retinal cells, like ganglion cells, retinal pigment epithelium (RPE) cells, or photoreceptors. The cells are sent to the retina, where they join with damaged tissue to aid in its healing. Then, the cells resume normal functioning. To encourage the growth of new native retinal cells, gene therapy or cell-based treatments can also be used. The treatment tries to slow down or stop the worsening of degenerative eye diseases like retinitis pigmentosa or AMD, which should improve the function of the retina and possibly restore vision. This method could be helpful in healing retinal diseases where other methods do not work very well [69–71]

The response to SCI depends critically on microglia, the resident immune cells of the CNS. They participate in the pathological mechanisms that impede regeneration, including scar development and inflammation. A possible approach to improve microglia’s neuroprotective and neurodegenerative properties is through epigenetic reprogramming. Veremeyko et al. are experiencing how neural elements might affect microglia’s epigenetic reprogramming in both standard and pathological conditions. They proposed that by altering the epigenetic environment of microglia, one can shift their function from neurotoxic to neurodegenerative [49] [67] [68]. Although the CNS exhibits limited regenerative capacity, certain forms of neuroplasticity and compensatory circuit remodeling can partially restore function following injury. Epigenome editing can help transplanted cells grow and heal by changing how they work to fit the microenvironment of the retina when used with these methods. This aids in the recovery and repair of degenerative retinal diseases (Fig. 5).

## Delivery Strategies for Epigenetic Editing

Practical therapeutic application requires not only precise editing tools but also reliable delivery systems capable of reaching target cells within the CNS. An exciting new approach for precisely altering gene expression without changing DNA sequence is epigenetic editing. Targeting DNA methylation, histone modifications, and ncRNAs, this approach is aimed at treating metabolic diseases, cancers, and neurological disorders. Viral vectors, particularly AAVs and lentiviruses, are increasingly utilized to deliver epigenetic editing tools due to their exceptional transduction capabilities and ability to sustain gene expression over an extended period [72] [73]. Engineered capsids (AAV-PHP.B/PHP.eB) significantly enhance CNS transduction in mice; however, cross-species translation has been variable, with specific variants exhibiting restricted efficacy in non-human primates. This highlights the importance of capsid evolution and thorough biodistribution assessment before clinical application [74].

People are interested in exosomes and lipid nanoparticles (LNPs) as safer and more flexible alternatives to viral vectors [75]. Because they can traverse the blood–brain barrier (BBB) and offer functional biomolecules, including proteins, RNAs, and small molecules, exosomes offer still another intriguing means of delivery for naturally occurring extracellular vesicles. Engineering exosomes loaded with epigenetic editing tools provides a biocompatible and least immunogenic method for focused gene control [76]. Scalability, purification, and loading efficiency must be addressed before exosome-based treatments can be broadly adopted.

The BBB remains one of the main obstacles to the methodical use of epigenetic editing technology. Highly selective, the BBB prevents most medicines and macromolecules from reaching the brain parenchyma. Techniques to get above this limit are receptor-mediated transcytosis (RMT), ultrasonic-assisted BBB disruption, and nanoparticle synthesis. Using endogenous transport systems, including low-density lipoprotein receptors or transferrin, RMT helps drugs be absorbed into the CNS. Engineering both viral and non-viral vectors has significantly improved BBB penetration, allowing for the exploitation of these pathways [77]. Focused ultrasonic (FUS) paired with microbubbles lowers systemic exposure and toxicity; therefore, momentarily upsetting the BBB and allowing therapeutic drugs to pass through [78]. It also relates to CNS-directed epigenetic editing—surface changes of nanoparticles that enhance BBB permeability and targeted administration, such as PEGylation or ligand conjugation. FU with microbubbles (FUS-BBBD) is advancing in human studies, offering a temporary, targetable approach to enhance the delivery of substantial biologics and gene editors to brain parenchyma, while current trials are fine-tuning safety and dose criteria [68] [79].

The systematic administration of epigenetic editing tools is still hampered in a significant part by the blood–brain barrier. Most macromolecules, including therapeutic medicines, cannot enter the brain parenchyma through the very selective endothelial interface of the BBB. RMT, ultrasonic-assisted BBB breakdown, and nanoparticle manufacturing are just a few of several creative approaches meant to overcome this obstacle. Table 1 presents a comparison of viral and non-viral epigenetic editing technologies.

**Table 1:** Contrasting viral and non-viral delivery systems for epigenetic editing:

Feature	Viral delivery (AAV, lentivirus)	Non-viral delivery (LNPs, exosomes)
Efficiency	High transduction efficiency	Lower efficiency, but improving with advanced formulations
Cargo capacity	Limited (AAV ~ 4.7 kb); lentivirus larger	Larger capacity, suitable for diverse payloads
Integration	AAV: episomal, lentivirus: genomic	Non-integrating, reducing the risk of insertional mutagenesis
Immunogenicity	Potential immune response	Generally lower immunogenicity
BBB penetration	AAV engineered for CNS delivery; lentivirus limited	LNPs and exosomes can cross the BBB with targeting modifications
Stability	Long-term expression possible	Shorter duration, often requiring repeated dosing
Safety concerns	Risk of insertional mutagenesis (lentivirus)	Lower genetic risks, but challenges in consistency and scalability
Manufacturing & scalability	Complex and costly	Easier to scale but requires optimization

## Challenges and Limitations

Despite remarkable progress, several technical and biological barriers still constrain the clinical translation of epigenetic editing. There are numerous benefits to epigenetic editing for controlling genes with precision; however, it requires further development before it can be applied in medicine. Unlike changes in genes, epigenetic changes can be reversed, influenced by environmental factors, cellular conditions, and disease progression. The duration of epigenetic modifications influences the persistence of therapeutic efficacy. To prolong the effects of epigenetic therapies, methods include repeated dosing, maintaining epigenetic marks, and harnessing cellular feedback networks, which are currently under research. Thus, further research is needed to elucidate the mechanisms regulating epigenetic memory and develop strategies for extending the duration of therapeutic alterations. Essential challenges in epigenetic editing also fall under ethical and safety concerns.

## Translational Barriers and Safety Considerations

### Off-Target Effects

Beyond mechanistic challenges, safety and translational feasibility remain central concerns that must be addressed before clinical implementation. One of the safety concerns of epigenetic editing is the accidental alterations to non-target genetic sites. This could be associated with an alteration in the gene expression profile or phenotypic abnormalities. Although CRISPR/dCas9 is one of the powerful technological tools that can precisely target specific genetic loci, it binds to off-target sites due to chromosomal accessibility, and the design of gRNA may be compromised [80]. This non-specific binding may target both coding and non-coding regulatory domains, and this potentially negatively affects essential cellular and molecular pathways. To mitigate these risks, orthogonal validation methods such as ChIP-seq and Assay for Transposase-Accessible Chromatin (ATAC-seq) [81], improved gRNA design algorithms, and high-fidelity Cas9 variants (eSpCas9 and HypaCas9) were employed. Nonetheless, a comprehensive preclinical investigation is requisite before advancing to clinical translation.

### Immunogenicity

Using viral vectors, such as lentiviruses and AAVs, may induce the host’s immune system to respond when they are used to deliver epigenetic editing tools into the host’s cells. Cas proteins from Streptococcus pyogenes can activate innate and adaptive immunity as they are considered foreign to the human immune system [82, 83]. This may not only reduce the effectiveness of gene delivery but also may trigger an inflammatory reaction that leads to the elimination of the editing machinery. Using humanized Cas9 or less immunogenic variations, and non-viral delivery methods such as LNPs or exosomes, may help overcome the immunogenicity issues associated with foreign antigens during the delivery process [83]. 

### Long-Term Stability of Edits

Epigenetic modifications, which are naturally reversible, can be affected by changes in cellular and environmental signals. Epigenetic plasticity helps avert irreversible modifications, although it may ultimately compromise therapeutic efficacy [84]. Researchers have discovered that epigenetic modifications, such as DNA demethylation and histone acetylation, may diminish over time, particularly in response to stress or cellular proliferation [84]. To extend the regulation of gene expression, contemporary strategies for enhancing the stability of therapeutic effects involve simultaneously addressing several chromatin layers (such as histone and DNA modifications) or administering editors frequently or as required. You could also strengthen the regulatory loops that get favorable input [30, 84]. You can potentially make the effects last longer by applying epigenetic editing on cells that do not divide, like neurons, to stop their spread [30, 84]. To promote the therapeutic application of epigenetic editing, it is crucial to address these translational challenges. To guarantee the safety and efficacy of next-generation epigenetic therapeutics, a comprehensive strategy is essential, emphasizing molecular precision, enhanced delivery, immunological safety, and stability of modifications. Further advancement of high-fidelity editors, non-immunogenic delivery systems, and long-term monitoring in preclinical models is essential to get from proof-of-concept to therapeutic use [54, 60, 85].

A preclinical study conducted in 2025 revealed that human PCSK9 may be successfully and permanently silenced in mouse models using a single LNP-delivered mRNA encoding a specified epigenetic editor. Tremblay and Xiong (2025) assert that this supports the concept of hit-and-run therapy and transient systemic injection techniques as advancements in epigenetic therapy [86].

## Future Perspectives and Clinical Translation

As these limitations are progressively overcome, the field is moving toward clinically viable strategies that redefine precision medicine. Epigenetic editing is a promising new tool that is expected to make precision medicine and personalized epigenetic therapy more accessible in the clinical setting soon, enabling the development of targeted therapeutic strategies tailored to each person’s unique genetic and epigenetic profiles. Utilizing recent technological advances, including multi-omics data, artificial intelligence (AI), and machine learning, promises to enhance the specificity and efficacy of epigenetic editing. These cutting-edge tools are developed not only to minimize off-target effects but also to enhance the duration of epigenetic modifications. Continuous improvements in gene delivery routes, combined with significant advancements in the field of synthetic biology, will lead to the application of epigenetic therapies in clinical settings. The utilization of multi-omics target discovery, AI-driven guide and effector design is expected to enhance the specificity and practicality of treatments [78]. Additionally, the success of durable in vivo editing (PCSK9) offers a substantial impetus for progress in the domain [87]. Currently, several clinical trials and regulatory paths are available due to the promising nature of epigenetic editing to provide safe and effective epigenetic treatments. Ethical and safety concerns relevant to epigenetic editing must be addressed as regulatory authorities establish guidelines for this technology. To maximize their impact on human health, forthcoming research should focus on improving long-term stability, ensuring equitable access to these novel therapies, and refining delivery mechanisms.

## Conclusion

Integrating the molecular, technological, and translational insights discussed above allows us to outline the overall promise and future direction of epigenetic editing in neurology. Studying epigenetic editing in neurological disorders reveals an exciting new area of precision medicine. A careful balance of epigenetic mechanisms that control gene expression is necessary for normal brain development, function, and plasticity. Different neurological disorders resulting from problems with these systems underscore the importance of epigenetic regulation in disease development and potential therapeutic approaches. CRISPR-based systems, histone modification techniques, and RNA-based methods offer unparalleled accuracy in modifying epigenetic markers and correcting aberrant gene expression patterns. These advancements provide a new framework for personalized therapies and substantial opportunities to create targeted medications that may tackle the underlying causes of neurodevelopmental and neurodegenerative disorders, even in their early stages. Although these treatments have considerable potential, it remains challenging to ensure they are both safe and effective, with long-term benefits. To address these challenges, enhance the tools for epigenetic editing, and apply these findings in therapeutic settings, we must continue to conduct research. Ongoing research may make epigenetic editing a promising new approach to treating neurological diseases. This could give people with hard-to-treat disorders a new sense of hope. 

## Data Availability

The paper and its supplementary information contain all the data supporting this review article.

## Abbreviations

AAV: Adeno-associated virus
AD: Alzheimer’s disease
AI: Artificial intelligence
AMD: Age-related macular degeneration
AS: Angelman syndrome
ASO: Antisense oligonucleotide
ATP: Adenosine triphosphate
BBB: Blood–brain barrier
BBV: Brain barrier vectors
BDNF: Brain-derived neurotrophic factor
BER: Base excision repair
Cas: CRISPR-associated protein
CNS: Central nervous system
CRISPR: Clustered regularly interspaced short palindromic repeats
dCas9: Catalytically dead Cas9
DNMT: DNA (cytosine-5)-methyltransferase
DNA: Deoxyribonucleic acid
FMR1: Fragile X messenger ribonucleoprotein 1
FMRP: Fragile X mental retardation protein
FUS: Focused ultrasound
FUS-BBBD: Focused ultrasound-mediated blood–brain barrier disruption
FXS: Fragile X syndrome
GABA: Gamma-aminobutyric acid
gRNA: Guide RNA
HAT: Histone acetyltransferase
HD: Huntington’s disease
HDAC: Histone deacetylase
iPSC: Induced pluripotent stem cell
KRAB: Krüppel-associated box
LNP: Lipid nanoparticle
lncRNA: Long non-coding RNA
MECP2: Methyl-CpG-binding protein 2
miRNA: MicroRNA
mRNA: Messenger RNA
ncRNA: Non-coding RNA
NDD: Neurodegenerative disease
NS/PC: Neural stem/progenitor cell
PD: Parkinson’s disease
PRC2: Polycomb repressive complex 2
RMT: Receptor-mediated transcytosis
RNA: Ribonucleic acid
RNAi: RNA interference
siRNA: Small interfering RNA
SWI/SNF: Switch/sucrose non-fermentable (chromatin remodeling complex)
TALEN: Transcription activator-like effector nuclease
TET: Ten-eleven translocation enzyme
TSHA-102: AAV9-based MECP2 gene therapy vector
UBE3A: Ubiquitin protein ligase E3A
ZFN: Zinc finger nuclease

## References

[1] Mehler MF (2008) Epigenetic principles and mechanisms underlying nervous system functions in health and disease. Prog Neurobiol 86(4):305–341 18940229 10.1016/j.pneurobio.2008.10.001 PMC2636693

[2] Millan MJ (2013) An epigenetic framework for neurodevelopmental disorders: from pathogenesis to potential therapy. Neuropharmacology 68:2–82 23246909 10.1016/j.neuropharm.2012.11.015

[3] Bashtrykov P, Jeltsch A (2017) Epigenome editing in the brain. Neuroepigenomics Aging Dis. 409–24. 10.1007/978-3-319-53889-1_21 28523558

[4] Ueda J, Yamazaki T, Funakoshi H (2023) Toward the development of epigenome editing-based therapeutics: potentials and challenges. Int J Mol Sci 24(5):4778 36902207 10.3390/ijms24054778 PMC10003136

[5] Li E, Zhang Y (2014) DNA methylation in mammals. Cold Spring Harb Perspect Biol 6(5):a019133 24789823 10.1101/cshperspect.a019133 PMC3996472

[6] Lyko F (2018) The DNA methyltransferase family: a versatile toolkit for epigenetic regulation. Nat Rev Genet 19(2):81–92 29033456 10.1038/nrg.2017.80

[7] Schübeler D (2015) Function and information content of DNA methylation. Nature 517(7534):321–326 25592537 10.1038/nature14192

[8] Jones PA (2012) Functions of DNA methylation: islands, start sites, gene bodies, and beyond. Nat Rev Genet 13(7):484–492 22641018 10.1038/nrg3230

[9] Lardenoije R, Iatrou A, Kenis G, Kompotis K, Steinbusch HW, Mastroeni D et al (2015) The epigenetics of aging and neurodegeneration. Prog Neurobiol 131:21–64 26072273 10.1016/j.pneurobio.2015.05.002 PMC6477921

[10] Bannister AJ, Kouzarides T (2011) Regulation of chromatin by histone modifications. Cell Res 21(3):381–395 21321607 10.1038/cr.2011.22 PMC3193420

[11] Tu J, Liao J, Luk ACS, Tang NLS, Chan WY, Lee TL (2015) Micrornas mediated targeting on the Yin-yang dynamics of DNA methylation in disease and development. Int J Biochem Cell Biol 67:115–120 25979370 10.1016/j.biocel.2015.05.002

[12] Policarpi C, Munafò M, Tsagkris S, Carlini V, Hackett JA (2024) Systematic epigenome editing captures the context-dependent instructive function of chromatin modifications. Nat Genet 56(6):1168–1180 38724747 10.1038/s41588-024-01706-w PMC11176084

[13] Thomson DW, Dinger ME (2016) Endogenous microRNA sponges: evidence and controversy. Nat Rev Genet 17(5):272–283 27040487 10.1038/nrg.2016.20

[14] Wu X, Zhang Y (2017) TET-mediated active DNA demethylation: mechanism, function and beyond. Nat Rev Genet 18(9):517–534 28555658 10.1038/nrg.2017.33

[15] Qian J, Liu SX (2024) CRISPR/dCas9-tet1-mediated DNA methylation editing. Bio-Protoc 14(8):e4976 38686348 10.21769/BioProtoc.4976 PMC11056002

[16] Yano N, Fedulov AV (2023) Targeted DNA demethylation: vectors, effectors and perspectives. Biomedicines 11(5):1334 37239005 10.3390/biomedicines11051334 PMC10215725

[17] Urbano A, Smith J, Weeks RJ, Chatterjee A (2019) Gene-specific targeting of DNA methylation in the mammalian genome. Cancers 11(10):1515 31600992 10.3390/cancers11101515 PMC6827012

[18] Maroufi F, Maali A, Abdollahpour-Alitappeh M, Ahmadi MH, Azad M (2020) CRISPR-mediated modification of DNA methylation pattern in the new era of cancer therapy. Epigenomics 12(20):1845–1859 33185489 10.2217/epi-2020-0110

[19] Kampmann M (2020) CRISPR-based functional genomics for neurological disease. Nat Rev Neurol 16(9):465–480 32641861 10.1038/s41582-020-0373-z PMC7484261

[20] Hilton IB, D’ippolito AM, Vockley CM, Thakore PI, Crawford GE, Reddy TE, et al. Epigenome editing by a CRISPR-Cas9-based acetyltransferase activates genes from promoters and enhancers. Nat Biotechnol. 2015;33(5):510–7. 10.1038/nbt.3199 PMC4430400 25849900

[21] Ma L, Yang S, Peng Q, Zhang J, Zhang J (2023) CRISPR/Cas9-based gene-editing technology for sickle cell disease. Gene 874:147480 37182559 10.1016/j.gene.2023.147480

[22] Zeng S, Lei S, Qu C, Wang Y, Teng S, Huang P (2023) CRISPR/Cas-based gene editing in therapeutic strategies for beta-thalassemia. Hum Genet 142(12):1677–1703 37878144 10.1007/s00439-023-02610-9

[23] Margolin JF, Friedman JR, Meyer W, Vissing H, Thiesen HJ, Rauscher F 3rd (1994) Krüppel-associated boxes are potent transcriptional repression domains. Proc Natl Acad Sci U S A 91(10):4509–4513 8183939 10.1073/pnas.91.10.4509 PMC43815

[24] Matsui T, Leung D, Miyashita H, Maksakova IA, Miyachi H, Kimura H et al (2010) Proviral silencing in embryonic stem cells requires the histone methyltransferase ESET. Nature 464(7290):927–931 20164836 10.1038/nature08858

[25] Gilbert LA, Larson MH, Morsut L, Liu Z, Brar GA, Torres SE et al (2013) CRISPR-mediated modular RNA-guided regulation of transcription in eukaryotes. Cell 154(2):442–451 23849981 10.1016/j.cell.2013.06.044 PMC3770145

[26] Gilbert LA, Horlbeck MA, Adamson B, Villalta JE, Chen Y, Whitehead EH et al (2014) Genome-scale CRISPR-mediated control of gene repression and activation. Cell 159(3):647–661 25307932 10.1016/j.cell.2014.09.029 PMC4253859

[27] Qi LS, Larson MH, Gilbert LA, Doudna JA, Weissman JS, Arkin AP et al (2013) Repurposing CRISPR as an RNA-guided platform for sequence-specific control of gene expression. Cell 152(5):1173–1183 23452860 10.1016/j.cell.2013.02.022 PMC3664290

[28] UE29.Yano N, Fedulov AV (2023) Targeted DNA demethylation: vectors, effectors and perspectives. Biomedicines. 11(5):1334. 10.3390/biomedicines11051334 PMC10215725 37239005

[29] Liu XS, Jaenisch R (2019) Editing the epigenome to tackle brain disorders. Trends Neurosci 42(12):861–870 31706628 10.1016/j.tins.2019.10.003

[30] Yeo NC, Chavez A, Lance-Byrne A, Chan Y, Menn D, Milanova D et al (2018) An enhanced CRISPR repressor for targeted mammalian gene regulation. Nat Methods 15(8):611–616 30013045 10.1038/s41592-018-0048-5 PMC6129399

[31] Nuñez JK, Chen J, Pommier GC, Cogan JZ, Replogle JM, Adriaens C et al (2021) Genome-wide programmable transcriptional memory by CRISPR-based epigenome editing. Cell 184(9):2503–2519 33838111 10.1016/j.cell.2021.03.025 PMC8376083

[32] Chen M, Zhu H, juanMao Y, Cao N, liYu Y, yunLi L et al (2020) Regulation of IL12B expression in human macrophages by TALEN-mediated epigenome editing. Curr Med Sci 40:900–909 33123904 10.1007/s11596-020-2249-2

[33] Vojta A, Dobrinić P, Tadić V, Bočkor L, Korać P, Julg B et al (2016) Repurposing the CRISPR-Cas9 system for targeted DNA methylation. Nucleic Acids Res 44(12):5615–5628 26969735 10.1093/nar/gkw159 PMC4937303

[34] Manickavinayaham S, Vélez-Cruz R, Biswas AK, Bedford E, Klein BJ, Kutateladze TG et al (2019) E2f1 acetylation directs p300/CBP-mediated histone acetylation at DNA double-strand breaks to facilitate repair. Nat Commun 10(1):4951 31666529 10.1038/s41467-019-12861-8 PMC6821830

[35] Nunna S (2015) Development of zinc finger methyltransferase fusion proteins for targeted DNA methylation and gene silencing in human cells

[36] Benedetti V, Banfi F, Zaghi M, Moll-Diaz R, Massimino L, Argelich L et al (2022) A SOX2-engineered epigenetic silencer factor represses the glioblastoma genetic program and restrains tumor development. Sci Adv 8(31):eabn3986 35921410 10.1126/sciadv.abn3986 PMC9348799

[37] Rinn JL, Kertesz M, Wang JK, Squazzo SL, Xu X, Brugmann SA et al (2007) Functional demarcation of active and silent chromatin domains in human HOX loci by noncoding RNAs. Cell 129(7):1311–1323 17604720 10.1016/j.cell.2007.05.022 PMC2084369

[38] Gupta RA, Shah N, Wang KC, Kim J, Horlings HM, Wong DJ et al (2010) Long non-coding RNA HOTAIR reprograms chromatin state to promote cancer metastasis. Nature 464(7291):1071–1076 20393566 10.1038/nature08975 PMC3049919

[39] Holoch D, Moazed D (2015) RNA-mediated epigenetic regulation of gene expression. Nat Rev Genet 16(2):71–84 25554358 10.1038/nrg3863 PMC4376354

[40] Riera M, Patel A, Corcostegui B, Chang S, Corneo B, Sparrow JR et al (2019) Generation of an induced pluripotent stem cell line (FRIMOi002-A) from a retinitis pigmentosa patient carrying compound heterozygous mutations in the USH2A gene. Stem Cell Res 35:101386 30685615 10.1016/j.scr.2019.101386

[41] Garzon R, Liu S, Fabbri M, Liu Z, Heaphy CE, Callegari E et al (2009) Microrna-29b induces global DNA hypomethylation and tumor suppressor gene reexpression in acute myeloid leukemia by targeting directly DNMT3A and 3B and indirectly DNMT1. Blood 113(25):6411–6418 19211935 10.1182/blood-2008-07-170589 PMC2710934

[42] Sato F, Tsuchiya S, Meltzer SJ, Shimizu K (2011) Micrornas and epigenetics. FEBS J 278(10):1598–1609 21395977 10.1111/j.1742-4658.2011.08089.x

[43] Kang JG, Park JS, Ko JH, Kim YS (2019) Regulation of gene expression by altered promoter methylation using a CRISPR/Cas9-mediated epigenetic editing system. Sci Rep 9(1):11960 31427598 10.1038/s41598-019-48130-3 PMC6700181

[44] Li X, Huang L, Pan L, Wang B, Pan L (2021) CRISPR/dCas9-mediated epigenetic modification reveals differential regulation of histone acetylation on *Aspergillus niger* secondary metabolite. Microbiol Res 245:126694 33482403 10.1016/j.micres.2020.126694

[45] Tan L, Yu JT, Hu N, Tan L (2013) Non-coding RNAs in Alzheimer’s disease. Mol Neurobiol 47:382–393 23054683 10.1007/s12035-012-8359-5

[46] Amabile A, Migliara A, Capasso P, Biffi M, Cittaro D, Naldini L et al (2016) Inheritable silencing of endogenous genes by hit-and-run targeted epigenetic editing. Cell 167(1):219–232 27662090 10.1016/j.cell.2016.09.006 PMC5039111

[47] Liyanage VR, Rastegar M (2014) Rett syndrome and MeCP2. Neuromolecular Med 16:231–264 24615633 10.1007/s12017-014-8295-9 PMC5798978

[48] Ure K, Lu H, Wang W, Ito-Ishida A, Wu Z, He L et al (2016) Restoration of Mecp2 expression in GABAergic neurons is sufficient to rescue multiple disease features in a mouse model of Rett syndrome. Elife 5:e14198 27328321 10.7554/eLife.14198 PMC4946897

[49] Coorey B, Haase F, Ellaway C, Clarke A, Lisowski L, Gold WA (2022) Gene editing and Rett syndrome: does it cut? CRISPR J 5(4):490–499 35881862 10.1089/crispr.2022.0020

[50] Ross PD, Gadalla KK, Thomson SR, Selfridge J, Bahey NG, Benito J et al (2025) Self-regulating gene therapy ameliorates phenotypes and overcomes gene dosage sensitivity in a mouse model of Rett syndrome. Sci Transl Med 17(792):eadq3614 40173263 10.1126/scitranslmed.adq3614

[51] Chiurazzi P, Pomponi MG, Willemsen R, Oostra BA, Neri G (1998) In vitro reactivation of the FMR1 gene involved in fragile X syndrome. Hum Mol Genet 7(1):109–113 9384610 10.1093/hmg/7.1.109

[52] Liu XS, Wu H, Krzisch M, Wu X, Graef J, Muffat J et al (2018) Rescue of fragile X syndrome neurons by DNA methylation editing of the FMR1 gene. Cell 172(5):979–992 29456084 10.1016/j.cell.2018.01.012 PMC6375087

[53] Kam CW, Dumelie JG, Ciceri G, Yang WY, Disney MD, Studer L et al (2025) Sustained epigenetic reactivation in fragile X neurons with an RNA-binding small molecule. Genes 16(3):278 40149430 10.3390/genes16030278 PMC11942054

[54] Hilton IB, D’ippolito AM, Vockley CM, Thakore PI, Crawford GE, Reddy TE, et al (2015) Epigenome editing by a CRISPR-Cas9-based acetyltransferase activates genes from promoters and enhancers. Nat Biotechnol. 33(5):510–7. 10.1038/nbt.3199 PMC4430400 25849900

[55] Qian J, Guan X, Xie B, Xu C, Niu J, Tang X et al (2023) Multiplex epigenome editing of MECP2 to rescue Rett syndrome neurons. Sci Transl Med 15(679):eadd4666 36652535 10.1126/scitranslmed.add4666 PMC11975455

[56] Jagasia R, Bon C, Rasmussen SV, Badillo S, Tehler D, Buchy D, et al (2022) Angelman syndrome patient neuron screen identifies a potent and selective clinical ASO targeting UBE3A-ATS with long-lasting effect in cynomolgus monkey. bioRxiv. 06. 10.1093/nar/gkaf851 PMC12397906 40884397

[57] Dindot SV, Christian S, Murphy WJ, Berent A, Panagoulias J, Schlafer A et al (2023) An ASO therapy for Angelman syndrome that targets an evolutionarily conserved region at the start of the UBE3A-AS transcript. Sci Transl Med 15(688):eabf4077 36947593 10.1126/scitranslmed.abf4077

[58] Jagadeeswaran I, Oh J, Sinnett SE (2025) Preclinical milestones in MECP2 gene transfer for treating Rett syndrome. Dev Neurosci 47(2):147–156 38723617 10.1159/000539267 PMC11965835

[59] Gillmore JD, Gane E, Taubel J, Kao J, Fontana M, Maitland ML et al (2021) CRISPR-Cas9 in vivo gene editing for transthyretin amyloidosis. N Engl J Med 385(6):493–502 34215024 10.1056/NEJMoa2107454

[60] Frangoul H, Altshuler D, Cappellini MD, Chen YS, Domm J, Eustace BK et al (2021) CRISPR-Cas9 gene editing for sickle cell disease and β-thalassemia. N Engl J Med 384(3):252–260 33283989 10.1056/NEJMoa2031054

[61] Kantor B, Tagliafierro L, Gu J, Zamora ME, Ilich E, Grenier C et al (2018) Downregulation of SNCA expression by targeted editing of DNA methylation: a potential strategy for precision therapy in PD. Mol Ther 26(11):2638–2649 30266652 10.1016/j.ymthe.2018.08.019 PMC6224806

[62] Yoon HH, Ye S, Lim S, Jo A, Lee H, Hong F et al (2022) CRISPR-Cas9 gene editing protects from the A53T-SNCA overexpression-induced pathology of Parkinson’s disease *in vivo*. CRISPR J 5(1):95–108 35191750 10.1089/crispr.2021.0025

[63] Cappelluti MA, Mollica Poeta V, Valsoni S, Quarato P, Merlin S, Merelli I et al (2024) Durable and efficient gene silencing in vivo by hit-and-run epigenome editing. Nature 627(8003):416–423 38418872 10.1038/s41586-024-07087-8 PMC10937395

[64] Yim YY, Teague CD, Nestler EJ (2020) In vivo locus-specific editing of the neuroepigenome. Nat Rev Neurosci 21(9):471–484 32704051 10.1038/s41583-020-0334-y PMC7439525

[65] Miller BH, Wahlestedt C (2010) Microrna dysregulation in psychiatric disease. Brain Res 1338:89–99 20303342 10.1016/j.brainres.2010.03.035 PMC2891055

[66] Finelli MJ, Wong JK, Zou H (2013) Epigenetic regulation of sensory axon regeneration after spinal cord injury. J Neurosci 33(50):19664–19676 24336730 10.1523/JNEUROSCI.0589-13.2013 PMC3858634

[67] Garriga J, Laumet G, Chen SR, Zhang Y, Madzo J, Issa JPJ et al (2018) Nerve injury-induced chronic pain is associated with persistent DNA methylation reprogramming in the dorsal root ganglion. J Neurosci 38(27):6090–6101 29875269 10.1523/JNEUROSCI.2616-17.2018 PMC6031579

[68] Hudry E, Vandenberghe LH (2019) Therapeutic AAV gene transfer to the nervous system: a clinical reality. Neuron 101(5):839–862 30844402 10.1016/j.neuron.2019.02.017 PMC11804970

[69] Ben M’Barek K, Habeler W, Regent F, Monville C (2019) Developing cell-based therapies for RPE-associated degenerative eye diseases. Pluripotent Stem Cells Eye Dis Ther. 2019;55–97. 10.1007/978-3-030-28471-8_3 31654386

[70] Gonzalez-Cordero A, Kruczek K, Naeem A, Fernando M, Kloc M, Ribeiro J et al (2017) Recapitulation of human retinal development from human pluripotent stem cells generates transplantable populations of cone photoreceptors. Stem Cell Rep 9(3):820–837 10.1016/j.stemcr.2017.07.022 PMC5599247 28844659

[71] Maeda A, Mandai M, Takahashi M (2019) Gene and induced pluripotent stem cell therapy for retinal diseases. Annu Rev Genomics Hum Genet 20(1):201–216 31018110 10.1146/annurev-genom-083118-015043

[72] Milone MC, O’Doherty U (2018) Clinical use of lentiviral vectors. Leukemia 32(7):1529–1541 29654266 10.1038/s41375-018-0106-0 PMC6035154

[73] Wang JH, Gessler DJ, Zhan W, Gallagher TL, Gao G (2024) Adeno-associated virus as a delivery vector for gene therapy of human diseases. Signal Transduct Target Ther 9(1):78 38565561 10.1038/s41392-024-01780-w PMC10987683

[74] Hou X, Zaks T, Langer R, Dong Y (2021) Lipid nanoparticles for mRNA delivery. Nat Rev Mater 6(12):1078–1094 34394960 10.1038/s41578-021-00358-0 PMC8353930

[75] Elsharkasy OM, Nordin JZ, Hagey DW, de Jong OG, Schiffelers RM, Andaloussi SE et al (2020) Extracellular vesicles as drug delivery systems: Why and how? Adv Drug Deliv Rev 159:332–343 32305351 10.1016/j.addr.2020.04.004

[76] Deverman BE, Pravdo PL, Simpson BP, Kumar SR, Chan KY, Banerjee A et al (2016) Cre-dependent selection yields AAV variants for widespread gene transfer to the adult brain. Nat Biotechnol 34(2):204–209 26829320 10.1038/nbt.3440 PMC5088052

[77] Burgess A, Shah K, Hough O, Hynynen K (2015) Focused ultrasound-mediated drug delivery through the blood–brain barrier. Expert Rev Neurother 15(5):477–491 25936845 10.1586/14737175.2015.1028369 PMC4702264

[78] Durham PG, Butnariu A, Alghorazi R, Pinton G, Krishna V, Dayton PA (2024) Current clinical investigations of focused ultrasound blood-brain barrier disruption: a review. Neurotherapeutics 21(3):e00352 38636309 10.1016/j.neurot.2024.e00352 PMC11044032

[79] Peter CJ, Saito A, Hasegawa Y, Tanaka Y, Nagpal M, Perez G et al (2019) In vivo epigenetic editing of the Sema6a promoter reverses transcallosal dysconnectivity caused by the C11orf46/Arl14ep risk gene. Nat Commun 10(1):4112 31511512 10.1038/s41467-019-12013-y PMC6739341

[80] Tycko J, Myer VE, Hsu PD (2016) Methods for optimizing CRISPR-Cas9 genome editing specificity. Mol Cell 63(3):355–370 27494557 10.1016/j.molcel.2016.07.004 PMC4976696

[81] Wagner DL, Amini L, Wendering DJ, Burkhardt LM, Akyüz L, Reinke P et al (2019) High prevalence of *Streptococcus pyogenes* Cas9-reactive T cells within the adult human population. Nat Med 25(2):242–248 30374197 10.1038/s41591-018-0204-6

[82] Charlesworth CT, Deshpande PS, Dever DP, Camarena J, Lemgart VT, Cromer MK et al (2019) Identification of preexisting adaptive immunity to Cas9 proteins in humans. Nat Med 25(2):249–254 30692695 10.1038/s41591-018-0326-x PMC7199589

[83] Goldberg AD, Allis CD, Bernstein E (2007) Epigenetics: a landscape takes shape. Cell 128(4):635–638 17320500 10.1016/j.cell.2007.02.006

[84] Tremblay F, Xiong Q, Shah SS, Ko CW, Kelly K, Morrison MS et al (2025) A potent epigenetic editor targeting human PCSK9 for durable reduction of low-density lipoprotein cholesterol levels. Nat Med 31(4):1329–1338 39930141 10.1038/s41591-025-03508-x PMC12003160

[85] Daci R, Gray-Edwards H, Shazeeb MS, Vardar Z, Vachha B, Cataltepe OI et al (2024) Neuroimaging applications for the delivery and monitoring of gene therapy for central nervous system diseases. Hum Gene Ther 35(21–22):886–895 39323316 10.1089/hum.2024.057 PMC13175210

[86] Roth GV, Gengaro IR, Qi LS (2024) Precision epigenetic editing: technological advances, enduring challenges, and therapeutic applications. Cell Chem Biol 31(8):1422–1446 10.1016/j.chembiol.2024.07.007 PMC11799355 39137782

[87] Tsai SQ, Wyvekens N, Khayter C, Foden JA, Thapar V, Reyon D et al (2014) Dimeric CRISPR RNA-guided FokI nucleases for precise genome editing. Nat Biotechnol 32(6):569–576 24770325 10.1038/nbt.2908 PMC4090141

